# Genome-Wide Analysis of Positively Selected Genes in Seasonal and Non-Seasonal Breeding Species

**DOI:** 10.1371/journal.pone.0126736

**Published:** 2015-05-22

**Authors:** Yuhuan Meng, Wenlu Zhang, Jinghui Zhou, Mingyu Liu, Junhui Chen, Shuai Tian, Min Zhuo, Yu Zhang, Yang Zhong, Hongli Du, Xiaoning Wang

**Affiliations:** 1 School of Bioscience and Bioengineering, Guangdong Provincial Key Laboratory of Fermentation and Enzyme Engineering, South China University of Technology, Guangzhou, China; 2 Guangdong Key Laboratory of Laboratory Animals/Guangdong laboratory animals monitoring institution, Guangzhou, China; 3 Chinese PLA General Hospital, Beijing, China; 4 School of Life Sciences, Fudan University, Shanghai, China; 5 Institute of Biodiversity Science, Tibet University, Lhasa, China; Smithsonian Conservation Biology Institute, UNITED STATES

## Abstract

Some mammals breed throughout the year, while others breed only at certain times of year. These differences in reproductive behavior can be explained by evolution. We identified positively-selected genes in two sets of species with different degrees of relatedness including seasonal and non-seasonal breeding species, using branch-site models. After stringent filtering by sum of pairs scoring, we revealed that more genes underwent positive selection in seasonal compared with non-seasonal breeding species. Positively-selected genes were verified by cDNA mapping of the positive sites with the corresponding cDNA sequences. The design of the evolutionary analysis can effectively lower the false-positive rate and thus identify valid positive genes. Validated, positively-selected genes, including *CGA*, *DNAH1*, *INVS*, and *CD151*, were related to reproductive behaviors such as spermatogenesis and cell proliferation in non-seasonal breeding species. Genes in seasonal breeding species, including *THRAP3*, *TH1L*, and *CMTM6*, may be related to the evolution of sperm and the circadian rhythm system. Identification of these positively-selected genes might help to identify the molecular mechanisms underlying seasonal and non-seasonal reproductive behaviors.

## Introduction

The environment can influence gene evolution and thus animal behaviors, including reproduction-related behaviors. Some mammals can breed throughout the year, while others only breed successfully at certain times of year. Such animals are defined as non-seasonal and seasonal breeding species, respectively. Day length, temperature, and food supply can all influence the reproductive behavior of seasonal breeding species and subsequent survival of offspring [1]; if they breed too early, the growing offspring may be exposed to low temperatures and scarce resources, whereas late breeding limits the time available for reproductive behaviors and preparation for the following winter. Accurate timing is therefore an essential component of life-history strategies for organisms living in seasonal environments [2]. The different reproductive behaviors of seasonal and non-seasonal breeding species may result from natural selection pressures [3]. Both strategies benefit the respective species to survive by adaption of their breeding behaviors to the environment through their long evolutionary histories. Whole genome-wide analysis of genes that are positively selected in mammal lineages using the respective breeding strategies may help us to understand the mechanisms responsible for the divergent reproductive behaviors as a result of adaptive evolution.

Positive Darwinian selection of protein-coding genes is a major driving force for detecting adaptive evolution and species diversification. The modified version of the branch-site test (Model A) [4, 5] was designed to detect localized episodic bouts of positive selection that affect only a few amino acid residues in particular lineages. This test has been shown to be a reasonably powerful tool, and has been widely used to investigate the adaptive evolution of genes in many species [6–8].

However, alignment errors may influence the results of branch-site gene analysis in mammalian and vertebrate species. It is therefore necessary to use reliable alignment methods to reduce the incidence of false-positive results [9]. Although the aligner software PRANK [10, 11] cannot eliminate false-positive results, it is nonetheless more powerful than other aligners [9, 12] such as MUSCLE [13] and ClustalW [14]. In addition to misalignments in multiple sequences, other factors such as sequence errors, misassembly, and annotation mistakes also increase the incidence of falsely-identified positive selection [15, 16]. More stringent filters are needed to ensure that branch-site analysis has a low and acceptable false positive rate.

In this genome-wide study, we investigated the evolution of seasonal breeding strategies by identifying positively-selected genes in non-seasonal and seasonal breeding species using modified branch-site models. We established Distant-Species and Close-Species sets, each of which included seasonal and non-seasonal groups. We then identified positively-selected genes in these groups. PRANK (codon) software was used to align all the gene orthologs in the two gene sets. However, because PRANK generates a relatively high false-positive rate with the branch-site model, stringent filtering using sum of pairs (SP) [17, 18] scoring was used to remove potentially unreliable alignments generated by multiple sequence alignments. Sequence errors, misassembly, and annotation mistakes were also detected by cDNA mapping. Functional analysis of genes identified as positively-selected after this stringent filtering process might help us to understand the molecular mechanisms that determine non-seasonal and seasonal breeding.

## Materials and Methods

### Materials preparation

Five non-seasonal breeding species and five seasonal breeding species were chosen as the Distant-Species set. The five non-seasonal species included: human (*Homo sapiens*, GRCh37), chimpanzee (*Pan troglodytes*, CHIMP2.1) [19], cynomolgus monkey (or crab-eating macaque, *Macaca fascicularis*) [20], mouse (*Mus musculus*, NCBIM37) [21] and rat (*Rattus norvegicus*, RGSC3.4) [22]. The five seasonal breeding species were Indian rhesus monkey (*Macaca mulatta*, MMUL_1) [23], Chinese rhesus monkey (*M*. *mulatta lasiota*, CR) [24], dog (*Canis familiaris*, BROADD2)[25], horse (*Equus caballus*, EquCab2) [26] and rabbit (*Oryctolagus cuniculus*, oryCun2) [27]. The long lineages between species in the Distant-Species set means that behaviors may have changed back and forth between seasonal and non-seasonal breeding strategies several times, while the divergent sequences might influence the branch-site model analysis and generate false positives [28]. To address this problem, we also established a Close-Species set that only included closely-related, non-seasonal (human, gorilla (*Gorilla gorilla*, gorGor3.1) [29], chimpanzee, and cynomolgus monkey), and seasonal-breeding species (orangutan (*Pongo abelii*, PPYG2) [30], Indian rhesus monkey, Chinese rhesus monkey, and marmoset (*Callithrix jacchus*, C_jacchus3.2.1) [31]).

The protein-coding sequences for human, gorilla, chimpanzee, orangutan, Indian rhesus monkey, marmoset, mouse, rat, dog, horse, and rabbit were downloaded from the Ensembl database (version 64, Sep. 2011; http://www.ensembl.org/info/data/ftp/index.html) [32]. The sequences for cynomolgus monkey (http://climb.genomics.cn/10.5524/100003) and Chinese rhesus macaque (http://climb.genomics.cn/10.5524/100002) were provided by BGI [33]. The corresponding cDNA sequences used in the accuracy assessment were downloaded from NCBI. Detailed information on the cDNA sequences used in this study are listed in S1 Table.

### Calculating positively-selected sites

To identify 1:1 gene orthologs, human protein sequences were used to conduct BLAST [34] searches against other species sequences (blastp-F T-e 1e-5-m 8). It is difficult to select a set of transcripts to minimize alignment gaps and potential errors and thus false-positive branch-site test results [35]. In simple analyses in previous studies [6–8, 33, 36–41], the longest transcript for a given gene was chosen. Reciprocal searches were then performed for each species protein sequences relative to human protein sequences. In each search, pairwise sequences with identities <60% were excluded, and the highest hit for each query was retained to determine the pairwise orthologs between humans and other species.

Modified branch-site models [5] for adaptive evolution analysis used each species in one breeding series as the foreground species, and all the other species in that breeding series as background species. For example, to test for positive selection in humans in the Distant-Species set, the human branch was designated as the foreground branch, and the other five species in the seasonal breeding group were designated as background branches. Positive selection signals for all species were tested similarly.

Protein-coding sequences associated with the corresponding 1:1 gene orthologs were aligned using PRANK (codon). The corresponding gene-based phylogenetic trees were constructed using the maximum likelihood method in the PHYLIP 3.69 [42] software package, according to the tested aligned protein-coding sequences. The aligned protein-coding sequences and the corresponding phylogenetic trees were then used to analyze the adaptive evolution using the branch-site model in PAML’s codeML program [4]. Branch-site modified model A (model = 2, NSsits = 2) and the corresponding null model (model = 2, NSsits = 2, fix_omega = 1 and omega = 1) [5] were used to identify sequences under positive selection in both test sets of animals. Significance was calculated using the χ^2^ statistic, with one degree of freedom. Genes with p ≤0.01 were considered to be positively selected [5]. The p values were adjusted according to the FDR method (multiple testing correction with the method of Benjamini and Hochberg) [43] to allow for multiple testing, with a strict criterion of FDR <0.05. Positively-selected sites were obtained based on the Bayes Empirical Bayes (BEB) analysis [5], with a posterior probability >95%.

### Screening for valid positive sites by SP penalty scoring

To ensure the accuracy of the positive sites, extended sequences were extracted including 15 amino acids (45 base pairs) upstream and downstream from the positive sites. SP [17, 18] measurements were then performed for penalty scoring of the sequences in both streams. (1) Some of the positive sites were at the edge of the beginning or end of the gene and were not reached by the upstream or downstream sequences, and the penalty base score was set separately for both streams (regarded as S, S = 15/n, where n is equal to the number of amino acids in the upstream or downstream sequence). (2) Penalty scores added 0 point for each position in perfect alignment, while mismatched sites or gaps in the alignment were awarded penalty scores of minus S or 2S, respectively. (3) Penalty scores for the upstream and downstream sequences were calculated separately, and the total penalty scores were the sum of the upstream and downstream scores. (4) Average penalty scores were calculated as the final scores (average penalty score = total penalty score/N, where N is the number of sequences used in each alignment).

General and individual penalty scores were used. General penalty scores were equal to the sum of the penalty scores from each of the two compared species. For individual penalty scores, sequences with positive sites were compared with each of the other sequences used in the alignment in turn, and the total penalty scores were regarded as the individual penalty score.

Threshold values were set for general and individual penalty scores to filter sequences with valid positive sites. In this study, the threshold values for the general and individual penalty scores were −50 and −15, respectively. If both the general and individual penalty scores were greater than the threshold value, the sequences were filtered and the sites regarded as positive.

### Accuracy of positive sites according to cDNA sequences

Mistakes can occur during genome sequencing, sequence assembly, or gene annotation, and cDNA sequences can be used as references to assess the accuracy of the positive sites.

Corresponding cDNA sequences were first matched to the gene sequences using the function BLAST [34] (blastn-e 1e-10-a 4-m 8). cDNA sequences that included the positions corresponding to the positive sites were then filtered. Further analysis was conducted using MEGA5 [44]. The gene sequences and their corresponding cDNA sequences were then subjected to alignment analysis using the MUSCLE [13] function. If the nucleotide sequences of the positive sites were identical to those of the corresponding positions in the cDNA sequences, the positive sites were regarded as valid.

## Results

### Preliminary filtering of positively-selected genes using PRANK and branch-site model

Totals of 11,031 and 13,171 1:1 gene orthologs with >60% identities were filtered from the Distant- and Close-Species sets, respectively, by BLAST [34]. The corresponding protein sequences were used for subsequent alignments. The numbers of pairwise gene orthologs between humans and other species are listed in S2 Table. After alignment using PRANK (codon), 10,918 gene orthologs in the Distant-Species set and 12,485 in the Close-Species set were tested for positive selection signals using the codeML program in the PAML package [4], with the modified branch-site model [5]. Positively-selected genes in each species with a p value <0.01(comparing LRT, the likelihood ratio test, with the χ2 distribution) and with a false-discovery rate (FDR) <5% are shown in Table 1.

**Table 1:** Numbers of positively-selected genes under different filtering conditions.

Class	Distant-Species	χ2 test p<0.01	Correction FDR<0.05	SP score fitered Genes	Close-Species	χ2 test p<0.01	Correction FDR<0.05	SP score fitered Genes
**Non-seasonal**	Human	88	16	4	Human	116	20	4
	Chimpanzee	207	68	27	Gorilla	274	163	34
	Cynomolgus	113	62	27	Chimpanzee	289	117	48
	Mouse	228	15	4	Cynomolgus	266	159	69
	Rat	274	43	18				
	Mean	182	40.8	16	Mean	236.25	114.75	38.75
**Seasonal**	Indian rhesus	453	361	131	Orangutan	446	303	147
	Chinese rhesus	203	110	51	Indian rhesus	603	464	157
	Dog	499	158	54	Chinese rhesus	229	130	57
	Horse	463	157	55	Marmoset	688	314	107
	Rabbit	444	129	58				
	Mean	412.4	183	69.8	Mean	491.5	302.75	117

**Fig 1:**
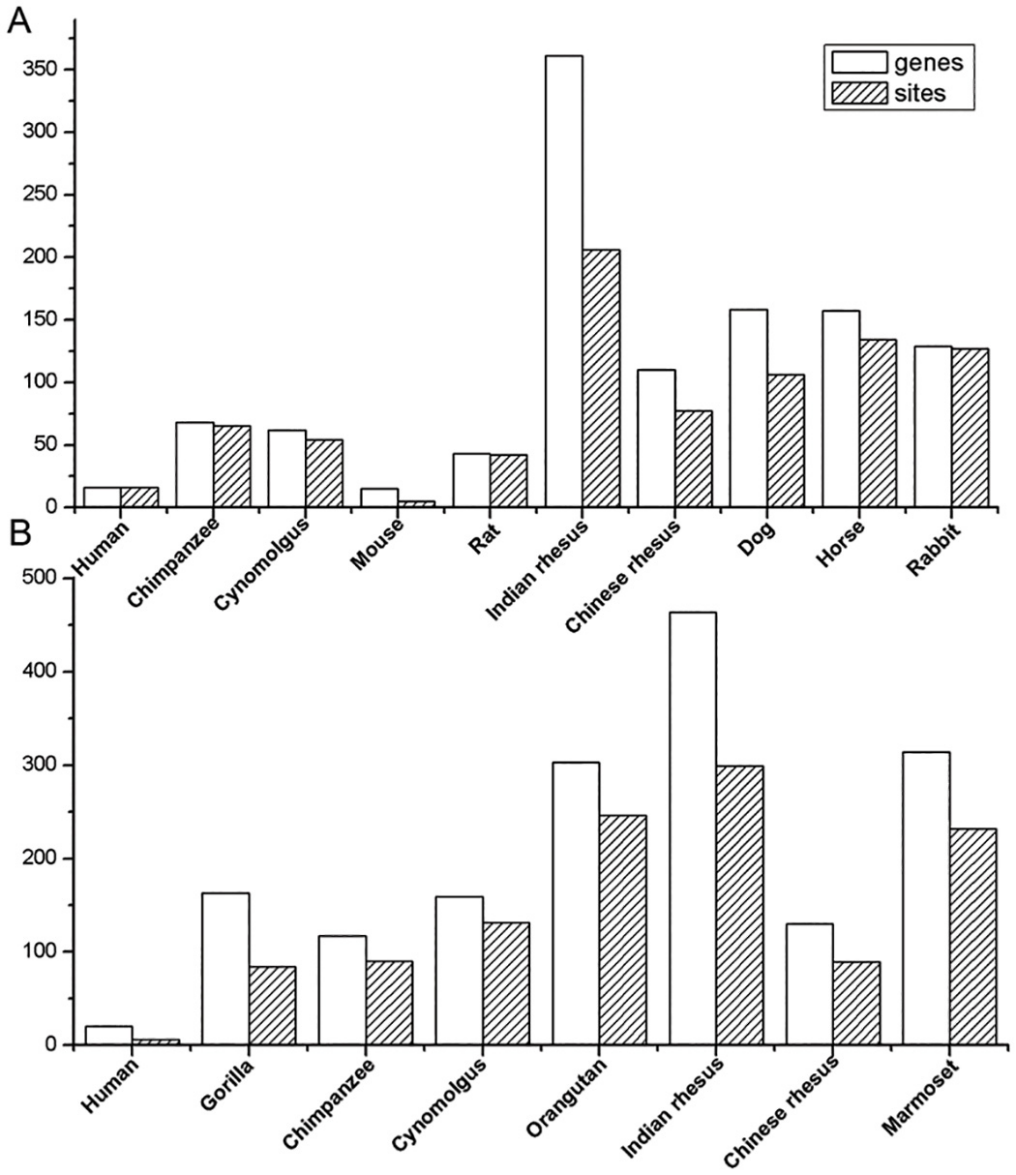
Numbers of positively-selectived genes (fdr <0.05) and sites (after SP-score filtering). (A). Positively-selected genes corrected by FDR. Sites (BEB >0.95) were filtered by SP scores in the Distant-Species set. (B). Positively-selected genes (FDR >0.05) and positive sites (BEB >0.95) filtered by general SP score >-50 and individual SP score >-15 in the Close-Species set.

In the Distant-Species set, the mean number of positively-selected genes in the seasonal species was four fold greater than in the non-seasonal species (fdr <0.05) (Fig 1A, Table 1). The equivalent increase in the Close-Species set was about 2.63-fold (Fig 1B, Table 1). These results demonstrate that there were more positively-selected genes in seasonal compared with non-seasonal breeders in both species sets.

However, there were more positively-selected genes in the Close-Species than in the Distant-Species set (mean numbers with FDR <0.05 208.75 and 111.9, respectively). In addition to the different numbers of orthologs (12,485 vs. 10,918), it is also possible that more gaps were generated by alignment in the Distant-Species gene ortholog set compared with in the Close-Species set (mean gap length 244 in the Close-Species set and 322 in the Distant-Species set) (S3 Table), because the sequence divergence was smaller in the Close-Species set. The number of gaps may influence the results of branch-site analysis, because the branch-site would remove columns with gaps in the alignment sequences and would thus exclude more potential positive sites in the Distant-Species set compared with the Close-Species set.

### Identification of false-positive sites through sequence misalignment

Putative positively-selected sites in the genome (FDR<0.05) were obtained by Bayes Empirical Bayes (BEB) analysis (posterior probability >95%) [5]. The numbers of putative positively-selected sites in each species are listed in Table 2. The details of all the positive sites with BEB >0.95 are listed in S4 Table.

**Table 2:** Positive sites after BEB and SP-score filtering.

Class	Distant-Species	Sites (BEB>0.95)	SP scores filtered sites	FPR	Close-Species	Sites (BEB>0.95)	SP scores filtered sites	FPR
**Non-seasonal**	Human	26	16	38.46%	Human	9	6	33.33%
	Chimpanzee	103	65	36.89%	Gorilla	158	84	46.84%
	Cynomolgus	92	54	41.30%	Chimpanzee	132	90	31.82%
	Mouse	10	5	50.00%	Cynomolgus	237	131	44.73%
	Rat	66	42	36.36%				
**Seasonal**	Indian rhesus	532	206	61.28%	Orangutan	444	246	44.59%
	Chinese rhesus	153	77	49.67%	Indian rhesus	531	299	43.69%
	Dog	261	106	59.39%	Chinese rhesus	189	89	52.91%
	Horse	262	134	48.85%	Marmoset	364	232	36.26%
	Rabbit	241	127	47.30%				

Alignment problems may influence the performance of the branch-site test, with poor alignment increasing the incidence of false-positive sites. We therefore filtered out sites with obvious signs of unreliable alignment. We also calculated the SP [17, 18] score for each of the positive sites’ extended sequences (± 15 amino acids/45 base pairs). Most unreliable alignments are represented by numerous gaps and sequence divergences (S1 Fig and S5 Table). After filtering, a total of 2009/3810 (52.73%) positive sites remained. Sites with extended alignments with low divergence are listed in S6 Table. The results after filtering revealed more sites with positive selection in the seasonal compared with the non-seasonal breeding species (Table 2). The false-positive rate due to misalignment was 33.33%–61.28% (Table 2), which was similar to that of 50%–55% in a previous report [12]. After alignment filtering, differences in gene numbers between species in the Distant- and Close-Species sets were consistent with those after FDR-adjusted filtering. However, the false positive rate(FPR) statistics only considered misalignment and did not take account of other factors such as sequence errors, misassembly, or annotation problems.

According to extended-sequence alignments of the positive sites, SP scores <-50 were generally caused by excessive gaps or deficient matches, of which gaps contributed more to the low SP penalty scores (S1 Fig and S6 Table). Gaps and deficient matches may arise as a result of diversity between species or different transcript lengths, because we used the longest human transcripts to BLAST other species’ protein-coding sequences [35]. Columns with gaps in the alignments would be deleted in branch-site models, even though positive sites may be located within deficient sequence alignments surrounded by gaps or mismatched sequences. A threshold SP score of −50 can filter out most false-positive sites caused by divergent sequence alignments. SP scoring thus improves the reliability of the results by reducing the false-positive rate caused by unreliable alignments. Details of the positive genes filtered by SP scoring are shown in S7 Table.

### cDNA mapping as a novel method of filtering positive sites

The quality of the genome may limit the accuracy of evolutionary analysis. It can result in false-positive results associated with sequencing errors, alternative splicing, amino acid repeats, and frameshift mutations, causing mistakes in gene annotation [8, 15]. However, cDNA sequences are much shorter than genome sequences and are thus more reliable. The reliability of positive sites will therefore be increased if sequences with positive sites are mapped to the corresponding cDNA sequences and aligned with most of the bases. We therefore used cDNA mapping as a novel means of testing sequence errors.

cDNA sequences corresponding to the positive sites were analyzed. In this study, we aligned a total of 193 positive sites in perfect alignment with at least one cDNA sequence of the corresponding species using the MUSCLE function [13] in MEGA5 [44]. The coverage between positive sites and corresponding cDNA sequences was low (<10%, 193/2009), and the false positive rate was 61.66% (120/193). Most inconsistent sites were in cynomolgus monkey, horse, and orangutan, which had genome sequences of low quality or with annotation mistakes. In contrast, the human, mouse and rat genome sequences showed high accuracy. The details of the positive sites mapped with the corresponding cDNA sequences are shown in S1 Table. A total of 74 corresponding cDNA sites were finally identified that were consistent with the positive sites (S1 Table). No corresponding cDNA sequences mapped to the positive sites in gorillas, Chinese rhesus monkeys, and marmosets. After verification by cDNA filtering, 39 genes remained, including 15 genes that were positively-selected in non-seasonal species (Table 3), and 24 in seasonal species (Table 4). Although the limited availability of cDNA sequences meant that only a few positive sites remained after mapping, these sites were likely to be more accurate.

**Table 3:** Positively-selected genes in non-seasonal species filtered by SP scoring and corrected by cDNA mapping.

Species	Gene Symbol	Species ID	Set	P-χ2 test	FDR correction
Human	*CGA*	ENST00000369582	Distant-Sspecies	0.000000	0.000779
Human	*TOMM6*	ENST00000398884	Distant-Sspecies	0.000046	0.035968
Human	*CD151*	ENST00000397420	Close-Sspecies	0.000045	0.029687
Human	*RRP8*	ENST00000254605	Distant-Sspecies	0.000040	0.033188
Human	*ACCN4*	ENST00000358078	Distant-Sspecies	0.000000	0.000808
			Close-Sspecies	0.000000	0.000609
Human	*CHRNA1*	ENST00000261007	Close-Sspecies	0.000000	0.000115
CE	*SNX5*	CE_ENSP00000366998	Distant-Sspecies	0.000000	0.000004
			Close-Sspecies	0.000000	0.000002
CE	*NCAPG*	CE_ENSP00000251496	Close-Sspecies	0.000013	0.002031
CE	*VPS33A*	CE_ENSP00000267199	Distant-Sspecies	0.000046	0.009533
Mouse	*SWI5*	ENSMUST00000113400	Distant-Sspecies	0.000032	0.032039
Mouse	*NID2*	ENSMUST00000022340	Distant-Sspecies	0.000005	0.005636
Mouse	*DHDH*	ENSMUST00000011526	Distant-Sspecies	0.000066	0.047987
Mouse	*DNAH1*	ENSMUST00000048603	Distant-Sspecies	0.000004	0.005318
Rat	*INVS*	ENSRNOT00000011622	Distant-Sspecies	0.000001	0.001202
Rat	*GALK2*	ENSRNOT00000012447	Distant-Sspecies	0.000146	0.037931

**Table 4:** Positively-selected genes in seasonal species filtered by SP scoring and corrected by cDNA mapping.

Species	Gene Symbol	Species ID	Set	P-χ2 test	FDR correction
Orangutan	*TADA1*	ENSPPYT00000000676	Close-Sspecies	0.000001	0.000150
Orangutan	*LGALS3BP*	ENSPPYT00000010154	Close-Sspecies	0.000000	0.000000
Orangutan	*ZFR*	ENSPPYT00000017875	Close-Sspecies	0.000000	0.000001
Orangutan	*THRAP3*	ENSPPYT00000001838	Close-Sspecies	0.000000	0.000001
Orangutan	*MTMR12*	ENSPPYT00000017872	Close-Sspecies	0.000000	0.000000
Orangutan	*TMCC2*	ENSPPYT00000000349	Close-Sspecies	0.000000	0.000018
Orangutan	*SLC44A2*	ENSPPYT00000011142	Close-Sspecies	0.000016	0.001520
Orangutan	*MIPEP*	ENSPPYT00000006166	Close-Sspecies	0.000010	0.001011
Orangutan	*XRN2*	ENSPPYT00000012494	Close-Sspecies	0.000000	0.000000
Orangutan	*RBM47*	ENSPPYT00000017075	Close-Sspecies	0.000000	0.000000
Orangutan	*MBTPS1*	ENSPPYT00000008921	Close-Sspecies	0.000138	0.008620
Orangutan	*FAM69A*	ENSPPYT00000001379	Close-Sspecies	0.000199	0.011474
Orangutan	*SLC43A2*	ENSPPYT00000009117	Close-Sspecies	0.000992	0.042126
Orangutan	*RAB1B*	ENSPPYT00000003634	Close-Sspecies	0.000026	0.002287
Orangutan	*CMTM6*	ENSPPYT00000016330	Close-Sspecies	0.000000	0.000003
Orangutan	*DARS2*	ENSPPYT00000000592	Close-Sspecies	0.000004	0.000458
Orangutan	*AARS*	ENSPPYT00000008865	Close-Sspecies	0.000002	0.000217
Orangutan	*TH1L*	ENSPPYT00000012980	Close-Sspecies	0.000000	0.000000
Rabbit	*PLEK*	ENSOCUT00000023428	Distant-Sspecies	0.000113	0.016744
Rabbit	*SNX25*	ENSOCUT00000024747	Distant-Sspecies	0.000005	0.002487
Dog	*ALB*	ENSCAFT00000037121	Distant-Sspecies	0.000019	0.004967
Horse	*SMC4*	ENSECAT00000024113	Distant-Sspecies	0.000108	0.015518
Horse	*ANO6*	ENSECAT00000013517	Distant-Sspecies	0.000007	0.003137
Horse	*GLIPR1*	ENSECAT00000016491	Distant-Sspecies	0.000035	0.007439

## Discussion

### Influence of alignment and annotation

The results of evolutionary analysis are influenced the quality of the genome sequence; false-positive sites may be detected and important information may be missed as a result of low-quality sequences [15, 16]. Unfortunately, recent genome-sequencing techniques are still unable to provide sequences reliable enough for evolutionary analysis. Stringent filtering functions and parameters are therefore needed to obtain reliable positive sites, and careful analytical design can achieve reliable results, even from low-quality genome sequences.

Evolutionary analysis usually starts with sequence alignment using software such as ClustalW, MUSCLE or PRANK. In this study, we used PRANK (codon), because this software takes evolutionary information into consideration before placing the gaps [11], resulting in fewer mismatches but larger gaps compared with the other programs (S3 Table). Valid positive sites are likely to be located in alignments with low divergence and few gaps or mismatches, and sequence misalignments can thus generate false-positive sites in branch-site models. The branch-site model usually deletes columns with gaps in the alignments when calculating positive sites, so some sites located in deficient alignments may be regarded as positive, whereas some true-positive sites may be missed. SP-score filtering, which focuses on filtering out such false-positive sites, can be used to reduce the false-positive rate and ensure the quality of the filtered positive sites. On the other hand, cDNA mapping can exclude false-positive sites that originate from mistakes in genome sequence assembly and gene annotation. The combination of these processes can thus filter out many false-positive sites and identify low-quality genome sequences, such as those for cynomolgus monkey, horse, and orangutan in this study.

cDNA sequences in previous genome-wide studies have generally been used as references for gene annotation [45–47]. In contrast, we used cDNA mapping as a novel method to identify positive sites with high quality. Because cDNA sequences are usually relatively short, current sequencing techniques can provide reliable sequences. Moreover, some sites can be mapped to more than one corresponding cDNA sequence. cDNA mapping can thus ensure the quality of the remaining positive sites. However, there are some limitations. More than 90% of sites cannot be matched with corresponding cDNA sequences, and the validity of these sites therefore cannot be checked using this method. Because cDNA sequences are usually sequenced for a specific purpose, corresponding cDNA sequences may not be available for some putative positive sites, and genes with important evolutionary implications may be missed.

### Positively-selected genes in seasonal and non-seasonal breeders

Evolutionary analysis of genome sequences can be used to identify specific, positively-selected genes in various species. The genetic mechanisms and potential environmental adaptations associated with seasonal and non-seasonal breeding can then be inferred by functional analysis of positively-selected genes in the respective species.

The functions of positively-selected genes in non-seasonal breeding species reflect reproductive tendencies such as sperm generation and cell proliferation. Two key genes perform these functions in humans: *CGA* (glycoprotein hormones, alpha polypeptide) is a gonadotropin subunit [48, 49], while *CD151* functions in promoting metastasis, and increases the expression of phospho-extracellular signal-regulated kinase (ERK) [50, 51]. Given that ERK is a component of the mitogen-activated protein kinase pathway, positive selection pressure on this gene may influence cell proliferation and differentiation [52, 53]. Mutation of *Dnah1* in mice has been reported to cause male infertility [54, 55], suggesting that it may play an important role in influencing mating behavior. Another crucial gene in rats, *Invs*, is involved in controlling cytoskeletal organization and cell division, which are essential for reproduction [56, 57]. Moreover, this gene can interact with NPHP1 and NPHP3 that influence the Wnt signaling pathway, which may in turn influence kidney function and renal cell formation linked to spermatocyte and spermatid generation in the testis [58–60]. These positively-selected genes may reflect modulation of the reproductive system under environmental pressure in non-seasonal breeding species, enabling them to breed throughout the year. The identification of positive sites focused on sperm generation and cell proliferation suggests that mutations in these genes may influence sperm quantity or reproductive capacity.

Genes that were positively selected in seasonal breeding species differed from those in non-seasonal species in having less focused functions. However, the orangutan provided the most valid positive genes among these species, and their functional analysis may help to explain some predominant characteristics of seasonal breeding species. The key gene, *THRAP3* (thyroid hormone receptor associated protein 3, also known as *Thrap150*), is a selective coactivator for CLOCK-BMAL1 and promotes CLOCK-BMAL1 binding to target genes [61]. Moreover, THRAP3 can also interact with HELZ2, which regulates adipocyte differentiation [62]. *Clock* and *Bmal1* have previously been reported to be closely related to seasonal breeding behaviors [63], the *THRAP3* mutation may thus influence the circadian rhythm of the reproductive system. This is supported by a previous study showing that thyroid hormone catabolism within the mediobasal hypothalamus regulated seasonal gonadotropin-releasing secretion [64]. However, because orangutans live in Indonesia, which has high temperature throughout the year [30, 65], they may not need to adjust their physical condition, such as lipid storage, to cope with cold weather. *THRAP3* may thus influence adipocyte differentiation, while other functionally-related genes such as *MTMR12* [66] and *ZFR* [67] would be positively selected because of such environmental conditions. In addition to *THRAP3*, the positively-selected genes *TH1L* and *CMTM6* may also help to explain the seasonal breeding behavior. As TH1L may have a similar function to TH1, which attenuates androgen signaling [68], while CMTM6 functions in spermatogenesis [69–71]. Evidence from previous studies suggests that orangutans produce 14 times less sperm than chimpanzees, which is a closely-related, but non-seasonal breeder [72]. Seasonal breeding in orangutans may thus be a consequence of circadian rhythm and limited sperm production, which restrict their breeding to the period from December to May, the most productive months in terms of food (fruit) supply, to ensure adequate food and energy for effective reproduction [73].

Diversity in breeding behaviors can generally be attributed to mutations affecting endocrine mechanisms. Such mutations may be related to specific environmental conditions, such as temperature and food supply. In this study, positively-selected genes related to sperm generation were identified in both types of breeding species. Indeed, previous reports have indicated rapid evolution of sperm proteins in mammals [74, 75]. Evolutionary mutations in these genes may not lead to the unique consequences associated with different breeding strategies. However, previous studies have indicated that the reproduction behavior in seasonal breeding species is largely under the regulation of the circadian rhythm system [64]. This is consistent with our results, which showed that *THRAP3*, which is functionally-related to the CLOCK-BMAL1 system, was under positive selection pressure. The mechanisms determining breeding behaviors can be complicated, but evolution leads to adaptation to the environment, enabling well-adapted lineages to persist for many generations.

## Conclusions

In this study, we conducted a precise, genome-wide scan to detect genes that were positively selected between seasonal and non-seasonal breeding species. The evolutionary analysis was designed to reduce the incidence of false-positive sites by SP filtering and cDNA mapping. Although the lack of cDNA sequences means that some positive genes may have been missed, the identification of valid, positively-selected genes with functions relating to spermatogenesis, cell proliferation, and circadian rhythm might indicate possible molecular mechanisms underlying the seasonal and non-seasonal reproductive behaviors. Further developments in genome-sequencing technologies will allow the sequencing and assembly of higher-quality genomes, and more accurate gene annotation, while the availability of more cDNA sequences will increase the value of cDNA mapping for improving the accuracy of evolutionary analysis.

## Supporting Information

S1 Fig Sites with extended sequences alignments. (A). Perfect alignment. (B). Acceptable alignment. (C). Unacceptable alignment because of large number of gaps. (D). Unacceptable alignment because of putative positive sites located in poorly-aligned sequences. (E). False negative. SP scoring filtered out mistaken acceptable alignments.
(TIF)

S1 Table Positive sites mapped with the corresponding cDNA sequences. (XLSX)

S2 Table 1:1 gene orthologs. Gene orthologs were generated by BLAST, and the best hit of human versus the other species was then reversed. All identities were >60%.
(XLSX)

S3 Table Lengths of gene sequences before and after alignments with different aligners. (XLSX)

S4 Table Positive sites (BEB >0.95). (XLSX)

S5 Table SP scores of positive sites after sequence alignment. (XLSX)

S6 Table Positive sites after SP-score filtering. (XLSX)

S7 Table Positive genes filtered by SP scoring. (XLSX)

## References

[1] Prendergast BJ. Internalization of seasonal time. Hormones and behavior. 2005;48(5):503–11. Epub 2005/07/20. 10.1016/j.yhbeh.2005.05.013 . 16026787

[2] Hut RA. Photoperiodism: shall EYA compare thee to a summer's day? Current biology: CB. 2011;21(1):R22–5. Epub 2011/01/11. 10.1016/j.cub.2010.11.060 . 21215931

[3] Ims RA. The ecology and evolution of reproductive synchrony. Trends in ecology & evolution. 1990;5(5):135–40. Epub 1990/05/01. 10.1016/0169-5347(90)90218-3 . 21232341

[4] Yang Z. PAML: a program package for phylogenetic analysis by maximum likelihood. Computer applications in the biosciences: CABIOS. 1997;13(5):555–6. Epub 1997/11/21. . 9367129 10.1093/bioinformatics/13.5.555

[5] Zhang J, Nielsen R, Yang Z. Evaluation of an improved branch-site likelihood method for detecting positive selection at the molecular level. Molecular biology and evolution. 2005;22(12):2472–9. Epub 2005/08/19. 10.1093/molbev/msi237 . 16107592

[6] Bakewell MA, Shi P, Zhang J. More genes underwent positive selection in chimpanzee evolution than in human evolution. Proceedings of the National Academy of Sciences of the United States of America. 2007;104(18):7489–94. Epub 2007/04/24. 10.1073/pnas.0701705104 17449636 PMC1863478

[7] Kosiol C, Vinar T, da Fonseca RR, Hubisz MJ, Bustamante CD, Nielsen R, et al Patterns of positive selection in six Mammalian genomes. PLoS genetics. 2008;4(8):e1000144 Epub 2008/08/02. 10.1371/journal.pgen.1000144 18670650 PMC2483296

[8] Sun YB, Zhou WP, Liu HQ, Irwin DM, Shen YY, Zhang YP. Genome-wide scans for candidate genes involved in the aquatic adaptation of dolphins. Genome biology and evolution. 2013;5(1):130–9. Epub 2012/12/19. 10.1093/gbe/evs123 23246795 PMC3595024

[9] Fletcher W, Yang Z. The effect of insertions, deletions, and alignment errors on the branch-site test of positive selection. Molecular biology and evolution. 2010;27(10):2257–67. Epub 2010/05/08. 10.1093/molbev/msq115 . 20447933

[10] Loytynoja A, Goldman N. An algorithm for progressive multiple alignment of sequences with insertions. Proceedings of the National Academy of Sciences of the United States of America. 2005;102(30):10557–62. Epub 2005/07/08. 10.1073/pnas.0409137102 16000407 PMC1180752

[11] Loytynoja A, Goldman N. Phylogeny-aware gap placement prevents errors in sequence alignment and evolutionary analysis. Science (New York, NY). 2008;320(5883):1632–5. Epub 2008/06/21. 10.1126/science.1158395 . 18566285

[12] Markova-Raina P, Petrov D. High sensitivity to aligner and high rate of false positives in the estimates of positive selection in the 12 Drosophila genomes. Genome research. 2011;21(6):863–74. Epub 2011/03/12. 10.1101/gr.115949.110 21393387 PMC3106319

[13] Edgar RC. MUSCLE: multiple sequence alignment with high accuracy and high throughput. Nucleic acids research. 2004;32(5):1792–7. Epub 2004/03/23. 10.1093/nar/gkh340 15034147 PMC390337

[14] Thompson JD, Gibson TJ, Higgins DG. Multiple sequence alignment using ClustalW and ClustalX. Current protocols in bioinformatics / editoral board, Andreas D Baxevanis [et al]. 2002;Chapter 2:Unit 2 3. Epub 2008/09/17. 10.1002/0471250953.bi0203s00 . 18792934

[15] Schneider A, Souvorov A, Sabath N, Landan G, Gonnet GH, Graur D. Estimates of positive Darwinian selection are inflated by errors in sequencing, annotation, and alignment. Genome biology and evolution. 2009;1:114–8. Epub 2009/01/01. 10.1093/gbe/evp012 20333182 PMC2817407

[16] Mallick S, Gnerre S, Muller P, Reich D. The difficulty of avoiding false positives in genome scans for natural selection. Genome research. 2009;19(5):922–33. Epub 2009/05/05. 10.1101/gr.086512.108 19411606 PMC2675981

[17] Altschul SF. Gap costs for multiple sequence alignment. Journal of theoretical biology. 1989;138(3):297–309. Epub 1989/06/08. . 2593679 10.1016/s0022-5193(89)80196-1

[18] Gupta SK, Kececioglu JD, Schaffer AA. Improving the practical space and time efficiency of the shortest-paths approach to sum-of-pairs multiple sequence alignment. Journal of computational biology: a journal of computational molecular cell biology. 1995;2(3):459–72. Epub 1995/01/01. . 8521275 10.1089/cmb.1995.2.459

[19] Mitani JC, Watts DP, Muller MN. Recent developments in the study of wild chimpanzee behavior. Evolutionary Anthropology: Issues, News, and Reviews. 2002;11(1):9–25. 10.1002/evan.10008

[20] Sun Z, Zeng L, Hong B, Zhang G. Primary Research on Reproduction of Cynomolgus Monkeys in an Indoor Breeding Mode in Beijing Area. Chinese Journal of Comparative Medicine. 2008;11:33–5.

[21] Bronson FH. The reproductive ecology of the house mouse. The Quarterly review of biology. 1979;54(3):265–99. Epub 1979/09/01. . 390600 10.1086/411295

[22] Perry JS. The Reproduction of the Wild Brown Rat (*Rattus norvegicus* Erxleben). Proceedings of the Zoological Society of London. 1945;115(1–2):19–46. 10.1111/j.1096-3642.1945.tb00849.x

[23] Harcourt AH, Harvey PH, Larson SG, Short RV. Testis weight, body weight and breeding system in primates. Nature. 1981;293(5827):55–7. Epub 1981/09/03. . 7266658 10.1038/293055a0

[24] Hou J, Qu W, Chen L, Zhang H. Study of the Reproduction Eco-Behavior of Macaca mulatta in Taihang Mountains. Chinese Journal of Ecology. 1998;17:22–5.

[25] Pal SK, Ghosh B, Roy S. Dispersal behaviour of free-ranging dogs (Canis familiaris) in relation to age, sex, season and dispersal distance. Applied Animal Behaviour Science. 1998;61(2):123–32. 10.1016/s0168-1591(98)00185-3

[26] Johnson L, Thompson DL Jr., Effect of seasonal changes in Leydig cell number on the volume of smooth endoplasmic reticulum in Leydig cells and intratesticular testosterone content in stallions. Journal of reproduction and fertility. 1987;81(1):227–32. Epub 1987/09/01. . 3668953 10.1530/jrf.0.0810227

[27] Brambell FWR. The Reproduction of the Wild Rabbit Oryctolagus cuniculus (L.). Proceedings of the Zoological Society of London. 1944;114(1–2):1–45. 10.1111/j.1096-3642.1944.tb00210.x

[28] Yang Z, dos Reis M. Statistical properties of the branch-site test of positive selection. Molecular biology and evolution. 2011;28(3):1217–28. Epub 2010/11/23. 10.1093/molbev/msq303 . 21087944

[29] Watts DP. Mountain gorilla reproduction and sexual behavior. American Journal of Primatology. 1991;24(3–4):211–25. 10.1002/ajp.1350240307 31952383

[30] Singleton I, van Schaik CP. The social organisation of a population of Sumatran orang-utans. Folia primatologica; international journal of primatology. 2002;73(1):1–20. Epub 2002/06/18. 10.1159/000060415 . 12065937

[31] Sousa MBC, Peregrino HPA, Cirne MFC, Mota MTS. Reproductive patterns and birth seasonality in a South-American breeding colony of common marmosets, *Callithrix jacchus* . Primates. 1999;40(2):327–36.

[32] Flicek P, Amode MR, Barrell D, Beal K, Brent S, Carvalho-Silva D, et al Ensembl 2012. Nucleic acids research. 2012;40(Database issue):D84–90. Epub 2011/11/17. 10.1093/nar/gkr991 22086963 PMC3245178

[33] Yan G, Zhang G, Fang X, Zhang Y, Li C, Ling F, et al Genome sequencing and comparison of two nonhuman primate animal models, the cynomolgus and Chinese rhesus macaques. Nature biotechnology. 2011;29(11):1019–23. Epub 2011/10/18. 10.1038/nbt.1992 . 22002653

[34] Altschul SF, Gish W, Miller W, Myers EW, Lipman DJ. Basic local alignment search tool. Journal of molecular biology. 1990;215(3):403–10. Epub 1990/10/05. 10.1016/s0022-2836(05)80360-2 . 2231712

[35] Villanueva-Canas JL, Laurie S, Alba MM. Improving genome-wide scans of positive selection by using protein isoforms of similar length. Genome biology and evolution. 2013;5(2):457–67. Epub 2013/02/05. 10.1093/gbe/evt017 23377868 PMC3590775

[36] Arbiza L, Dopazo J, Dopazo H. Positive selection, relaxation, and acceleration in the evolution of the human and chimp genome. PLoS computational biology. 2006;2(4):e38 Epub 2006/05/10. 10.1371/journal.pcbi.0020038 16683019 PMC1447656

[37] Rhesus Macaque Genome S, Analysis C, Gibbs RA, Rogers J, Katze MG, Bumgarner R, et al Evolutionary and biomedical insights from the rhesus macaque genome. Science (New York, NY). 2007;316(5822):222–34. Epub 2007/04/14. 10.1126/science.1139247 . 17431167

[38] Vilella AJ, Severin J, Ureta-Vidal A, Heng L, Durbin R, Birney E. EnsemblCompara GeneTrees: Complete, duplication-aware phylogenetic trees in vertebrates. Genome research. 2009;19(2):327–35. Epub 2008/11/26. 10.1101/gr.073585.107 19029536 PMC2652215

[39] Toll-Riera M, Laurie S, Alba MM. Lineage-specific variation in intensity of natural selection in mammals. Molecular biology and evolution. 2011;28(1):383–98. Epub 2010/08/07. 10.1093/molbev/msq206 . 20688808

[40] Carneiro M, Albert FW, Melo-Ferreira J, Galtier N, Gayral P, Blanco-Aguiar JA, et al Evidence for widespread positive and purifying selection across the European rabbit (Oryctolagus cuniculus) genome. Molecular biology and evolution. 2012;29(7):1837–49. Epub 2012/02/10. 10.1093/molbev/mss025 22319161 PMC3375474

[41] Laurie S, Toll-Riera M, Rado-Trilla N, Alba MM. Sequence shortening in the rodent ancestor. Genome research. 2012;22(3):478–85. Epub 2011/12/01. 10.1101/gr.121897.111 22128134 PMC3290783

[42] Felsenstein J. PHYLIP: Phylogeny Inference Package. University of Washington,Seattle, WA (http://evolution.genetics.washington.edu/phylip.html). 1993.

[43] Benjamini Y, Hochberg Y. Controlling the false discovery rate: a practical and powerful approach to multiple testing. Journal of the Royal Statistical Society Series B (Methodological). 1995:289–300.

[44] Tamura K, Peterson D, Peterson N, Stecher G, Nei M, Kumar S. MEGA5: molecular evolutionary genetics analysis using maximum likelihood, evolutionary distance, and maximum parsimony methods. Molecular biology and evolution. 2011;28(10):2731–9. Epub 2011/05/07. 10.1093/molbev/msr121 21546353 PMC3203626

[45] Furuno M, Kasukawa T, Saito R, Adachi J, Suzuki H, Baldarelli R, et al CDS annotation in full-length cDNA sequence. Genome research. 2003;13(6B):1478–87. Epub 2003/06/24. 10.1101/gr.1060303 12819146 PMC403693

[46] Kim DS, Huh JW, Kim YH, Park SJ, Lee SR, Chang KT. Full-length cDNA sequences from Rhesus monkey placenta tissue: analysis and utility for comparative mapping. BMC genomics. 2010;11:427 Epub 2010/07/14. 10.1186/1471-2164-11-427 20624290 PMC2996955

[47] Uenishi H, Morozumi T, Toki D, Eguchi-Ogawa T, Rund LA, Schook LB. Large-scale sequencing based on full-length-enriched cDNA libraries in pigs: contribution to annotation of the pig genome draft sequence. BMC genomics. 2012;13:581 Epub 2012/11/16. 10.1186/1471-2164-13-581 23150988 PMC3499286

[48] Rebers F, Tensen C, Schulz R, Goos HT, Bogerd J. Modulation of glycoprotein hormone α-and gonadotropin IIβ-subunit mRNA levels in the pituitary gland of mature male African catfish, Clarias gariepinus. Fish Physiology and Biochemistry. 1997;17(1–6):99–108.

[49] Orth JM. Cell biology of testicular development in the fetus and neonate. Cell and molecular biology of the testis. 1993:3–42.

[50] Yang W, Li P, Lin J, Zuo H, Zuo P, Zou Y, et al CD151 promotes proliferation and migration of PC3 cells via the formation of CD151-integrin alpha3/alpha6 complex. Journal of Huazhong University of Science and Technology Medical sciences = Hua zhong ke ji da xue xue bao Yi xue Ying De wen ban = Huazhong keji daxue xuebao Yixue Yingdewen ban. 2012;32(3):383–8. Epub 2012/06/12. 10.1007/s11596-012-0066-y . 22684562

[51] Yue S, Mu W, Zoller M. Tspan8 and CD151 promote metastasis by distinct mechanisms. European journal of cancer (Oxford, England: 1990). 2013;49(13):2934–48. Epub 2013/05/21. 10.1016/j.ejca.2013.03.032 . 23683890

[52] Jang YN, Baik EJ. JAK-STAT pathway and myogenic differentiation. Jak-Stat. 2013;2(2):e23282 Epub 2013/09/24. 10.4161/jkst.23282 24058805 PMC3710318

[53] Weber-Nordt RM, Mertelsmann R, Finke J. The JAK-STAT pathway: signal transduction involved in proliferation, differentiation and transformation. Leukemia & lymphoma. 1998;28(5–6):459–67. Epub 1998/06/05. 10.3109/10428199809058353 . 9613975

[54] Ben Khelifa M, Coutton C, Zouari R, Karaouzene T, Rendu J, Bidart M, et al Mutations in DNAH1, which encodes an inner arm heavy chain dynein, lead to male infertility from multiple morphological abnormalities of the sperm flagella. American journal of human genetics. 2014;94(1):95–104. Epub 2013/12/24. 10.1016/j.ajhg.2013.11.017 24360805 PMC3882734

[55] Neesen J, Kirschner R, Ochs M, Schmiedl A, Habermann B, Mueller C, et al Disruption of an inner arm dynein heavy chain gene results in asthenozoospermia and reduced ciliary beat frequency. Human molecular genetics. 2001;10(11):1117–28. Epub 2001/05/24. . 11371505 10.1093/hmg/10.11.1117

[56] Veland IR, Montjean R, Eley L, Pedersen LB, Schwab A, Goodship J, et al Inversin/Nephrocystin-2 is required for fibroblast polarity and directional cell migration. PloS one. 2013;8(4):e60193 Epub 2013/04/18. 10.1371/journal.pone.0060193 23593172 PMC3620528

[57] Werner ME, Ward HH, Phillips CL, Miller C, Gattone VH, Bacallao RL. Inversin modulates the cortical actin network during mitosis. American journal of physiology Cell physiology. 2013;305(1):C36–47. Epub 2013/03/22. 10.1152/ajpcell.00279.2012 23515530 PMC3725518

[58] Lienkamp S, Ganner A, Walz G. Inversin, Wnt signaling and primary cilia. Differentiation; research in biological diversity. 2012;83(2):S49–55. Epub 2011/12/31. 10.1016/j.diff.2011.11.012 . 22206729

[59] Benzing T, Simons M, Walz G. Wnt signaling in polycystic kidney disease. Journal of the American Society of Nephrology: JASN. 2007;18(5):1389–98. Epub 2007/04/13. 10.1681/asn.2006121355 . 17429050

[60] Nurnberger J, Kavapurackal R, Zhang SJ, Opazo Saez A, Heusch G, Philipp T, et al Differential tissue distribution of the Invs gene product inversin. Cell and tissue research. 2006;323(1):147–55. Epub 2005/07/12. 10.1007/s00441-005-0012-4 . 16007506

[61] Lande-Diner L, Boyault C, Kim JY, Weitz CJ. A positive feedback loop links circadian clock factor CLOCK-BMAL1 to the basic transcriptional machinery. Proceedings of the National Academy of Sciences of the United States of America. 2013;110(40):16021–6. Epub 2013/09/18. 10.1073/pnas.1305980110 24043798 PMC3791755

[62] Katano-Toki A, Satoh T, Tomaru T, Yoshino S, Ishizuka T, Ishii S, et al THRAP3 interacts with HELZ2 and plays a novel role in adipocyte differentiation. Molecular endocrinology (Baltimore, Md). 2013;27(5):769–80. Epub 2013/03/26. 10.1210/me.2012-1332 . 23525231 PMC5416755

[63] Lincoln GA, Andersson H, Loudon A. Clock genes in calendar cells as the basis of annual timekeeping in mammals—a unifying hypothesis. The Journal of endocrinology. 2003;179(1):1–13. Epub 2003/10/08. . 14529560 10.1677/joe.0.1790001

[64] Yoshimura T. Neuroendocrine mechanism of seasonal reproduction in birds and mammals. Animal science journal = Nihon chikusan Gakkaiho. 2010;81(4):403–10. Epub 2010/07/29. 10.1111/j.1740-0929.2010.00777.x . 20662808

[65] Rijksen HD, Wageningen L. A fieldstudy on Sumatran orang utans (Pongo pygmaeus abelii, Lesson 1827): Ecology, behaviour and conservation: H. Veenman Netherlands; 1978.

[66] Gupta VA, Hnia K, Smith LL, Gundry SR, McIntire JE, Shimazu J, et al Loss of catalytically inactive lipid phosphatase myotubularin-related protein 12 impairs myotubularin stability and promotes centronuclear myopathy in zebrafish. PLoS genetics. 2013;9(6):e1003583 Epub 2013/07/03. 10.1371/journal.pgen.1003583 23818870 PMC3688503

[67] Prorocic MM, Wenlong D, Olorunniji FJ, Akopian A, Schloetel JG, Hannigan A, et al Zinc-finger recombinase activities in vitro. Nucleic acids research. 2011;39(21):9316–28. Epub 2011/08/19. 10.1093/nar/gkr652 21849325 PMC3241657

[68] Yang Y, Zou W, Kong X, Wang H, Zong H, Jiang J, et al Trihydrophobin 1 attenuates androgen signal transduction through promoting androgen receptor degradation. Journal of cellular biochemistry. 2010;109(5):1013–24. Epub 2010/01/14. 10.1002/jcb.22484 . 20069563

[69] Stittrich AB, Haftmann C, Sgouroudis E, Kuhl AA, Hegazy AN, Panse I, et al The microRNA miR-182 is induced by IL-2 and promotes clonal expansion of activated helper T lymphocytes. Nature immunology. 2010;11(11):1057–62. Epub 2010/10/12. 10.1038/ni.1945 . 20935646

[70] Zhong W-d, Zeng G-q, Cai Y-b, Tan Y, Hen S, Dai Q, et al Pathological changes in seminiferous tubules in infertility rats induced by chemokine-like factor I. Chin J Exp Surg. 2003;20:1027–8.

[71] Liu D, Yin C, Zhang Y, Tian L, Li T, Li D, et al Human CMTM2/CKLFSF2 enhances the ligand-induced transactivation of the androgen receptor. Chinese Science Bulletin. 2009;54(6):1050–7.

[72] Fujii-Hanamoto H, Matsubayashi K, Nakano M, Kusunoki H, Enomoto T. A comparative study on testicular microstructure and relative sperm production in gorillas, chimpanzees, and orangutans. Am J Primatol. 2011;73(6):570–7. Epub 2011/02/03. 10.1002/ajp.20930 . 21287585

[73] Wich SA, Utami-Atmoko SS, Setia TM, Rijksen HD, Schurmann C, van Hooff JA, et al Life history of wild Sumatran orangutans (Pongo abelii). Journal of human evolution. 2004;47(6):385–98. Epub 2004/11/30. 10.1016/j.jhevol.2004.08.006 . 15566945

[74] Torgerson DG, Kulathinal RJ, Singh RS. Mammalian sperm proteins are rapidly evolving: evidence of positive selection in functionally diverse genes. Molecular biology and evolution. 2002;19(11):1973–80. Epub 2002/11/02. . 12411606 10.1093/oxfordjournals.molbev.a004021

[75] Swanson WJ, Vacquier VD. The rapid evolution of reproductive proteins. Nature reviews Genetics. 2002;3(2):137–44. Epub 2002/02/12. 10.1038/nrg733 . 11836507

