# Supplementary figures and images for: Genome-Wide Analysis of Positively Selected Genes in Seasonal and Non-Seasonal Breeding Species
Source: PLoS One. 2015 May 22;10(5):e0126736. doi: 10.1371/journal.pone.0126736 (PMC4441472; doi:10.1371/journal.pone.0126736)

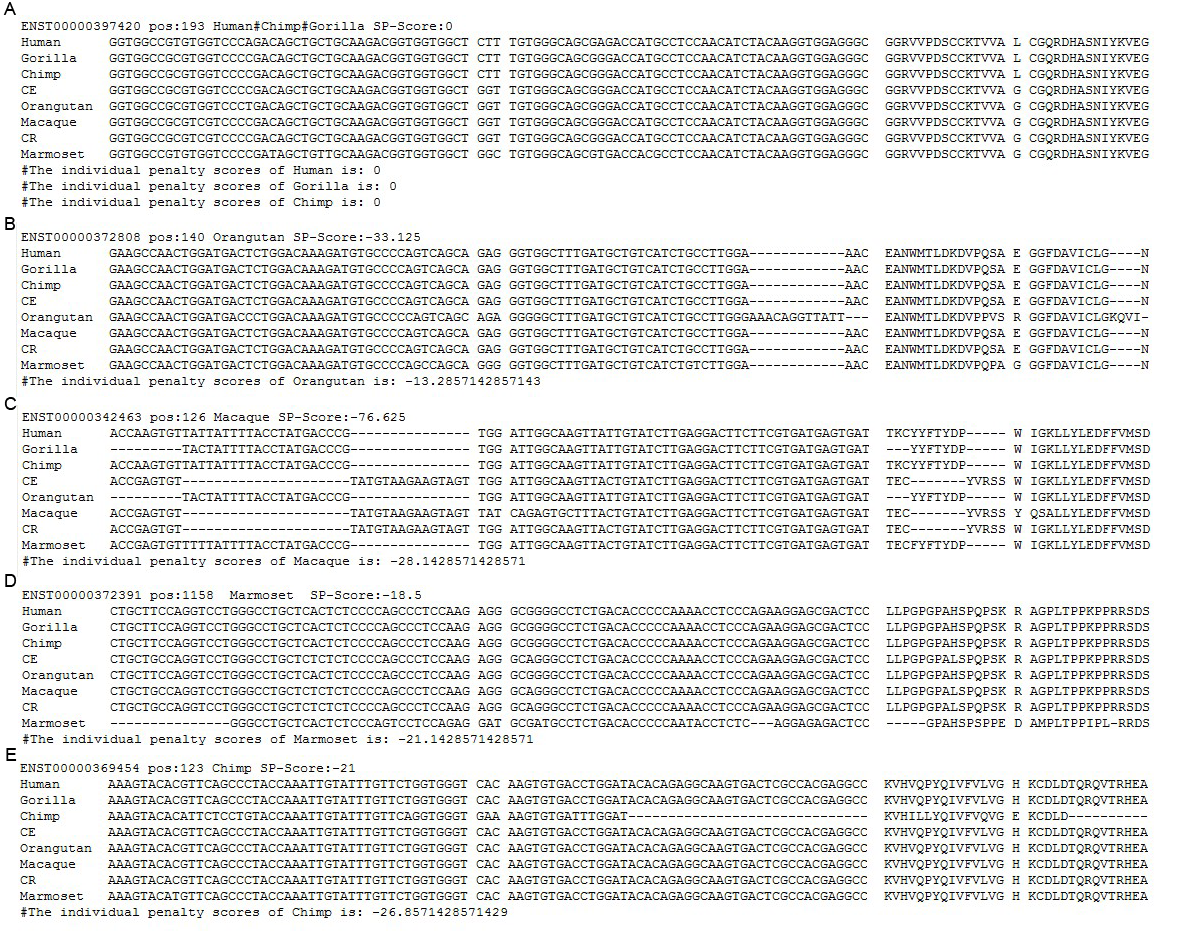

Supplement: S1 Fig — (A). Perfect alignment. (B). Acceptable alignment. (C). Unacceptable alignment because of large number of gaps. (D). Unacceptable alignment because of putative positive sites located in poorly-aligned sequences. (E). False negative. SP scoring filtered out mistaken acceptable alignments. (TIF) [file pone.0126736.s001.tif]
